# COVID-19 and Sepsis

**DOI:** 10.3906/sag-2108-239

**Published:** 2021-12-17

**Authors:** Zeliha KOÇAK TUFAN, Bircan KAYAASLAN, Mervyn MER

**Affiliations:** 1 Department of Infectious Disease and Clinical Microbiology, Ankara Yildirim Beyazit University, Ankara City Hospital, Ankara Turkey; 2 Executive Board Member of Council of Higher Education of Turkey (YÖK; 3 Member of COVID-19 Advisory Committee of Ministry of Health of Turkey; 4 Divisions of Critical Care and Pulmonology, Department of Medicine, Charlotte Maxeke Johannesburg Academic Hospital and Faculty of Health Sciences University of the Witwatersrand, Johannesburg South Africa

**Keywords:** COVID-19, sepsis, multiorgan failure, dysregulated immune response, hyperinflammation, cytokine storm

## Abstract

The COVID-19 pandemic has created a major alteration in the medical literature including the sepsis discussion. From the outset of the pandemic, various reports have indicated that although there are some unique features pertinent to COVID-19, many of its acute manifestations are similar to sepsis caused by other pathogens. As a consequence, the old definitions now require consideration of this new etiologic agent, namely SARS-CoV-2. Although the pathogenesis of COVID-19 has not been fully explained, the data obtained so far in hospitalized patients has revealed that serum cytokine and chemokine levels are high in severe COVID-19 patients, similar to those found with sepsis. COVID-19 may involve multiple organ systems. In addition to the lungs, the virus has been isolated from blood, urine, faeces, liver, and gallbladder. Results from autopsy series in COVID-19 patients have demonstrated a wide range of findings, including vascular involvement, congestion, consolidation, and hemorrhage as well as diffuse alveolar damage in lung tissue consistent with acute respiratory distress syndrome (ARDS). The presence of viral cytopathic-like changes, infiltration of inflammatory cells (mononuclear cells and macrophages), and viral particles in histopathological samples are considered a consequence of both direct viral infection and immune hyperactivation. Thromboembolism and hyper-coagulopathy are other components in the pathogenesis of severe COVID-19. Although the pathogenesis of hypercoagulability is not fully understood, it has been pointed out that all three components of Virchow’s triad (endothelial injury, stasis, and hypercoagulable state) play a major role in contributing to clot formation in severe COVID-19 infection. In severe COVID-19 cases, laboratory parameters such as hematological findings, coagulation tests, liver function tests, D-dimer, ferritin, and acute phase reactants such as CRP show marked alterations, which are suggestive of a cytokine storm. Another key element of COVID-19 pathogenesis in severe cases is its similarity or association with hemophagocytic lymphohistiocytosis (HLH). SARS-CoV-2 induced cytokine storm has significant clinical and laboratory findings overlapping with HLH. Viral sepsis has some similarities but also some differences when compared to bacterial sepsis. In bacterial sepsis, systemic inflammation affecting multiple organs is more dominant than in COVID-19 sepsis. While bacterial sepsis causes an early and sudden onset clinical deterioration, viral diseases may exhibit a relatively late onset and chronic course. Consideration of severe COVID-19 disease as a sepsis syndrome has relevance and may assist in terms of determining treatments that will modulate the immune response, limit intrinsic damage to tissue and organs, and potentially improve outcome.

## 1. Introduction 

Sepsis is the most common cause of emergency admission to the intensive care unit (ICU) and one of the major reasons for death among hospitalized patients in ICU [1,2]. The first sepsis definition was introduced in the early 1990s, and it has been updated over time by Surviving Sepsis Campaign (SSC) [3]. The current definition alludes to sepsis as a life-threatening organ dysfunction caused by a dysregulated host response to infection. However, the definition of sepsis is a complex issue, and it should be emphasized that sepsis is a clinical syndrome that is determined by the characteristics of both causative pathogens and patients (including such variables as age, gender, genetics, and underlying disease). The terminology regarding sepsis is changing over time and is likely to further evolve based on new insights, as well as the detection of new pathogens and diagnostic tools. Although sepsis can occur as a result of a viral etiology, viruses have not been as prominently implicated in the etiology as have other pathogens such as bacteria and fungi, until recently. The COVID-19 pandemic has created a major alteration in the medical literature including the sepsis discussion. Therefore, the old definitions now require consideration of this new etiologic agent, namely SARS-CoV-2.

The current definition of sepsis is focused on the inflammatory basis of the disease. Accumulating data on the pathophysiology of sepsis emphasizes that sepsis results from an imbalanced host reaction between the proinflammatory and antiinflammatory response to infection [4,5]. Although the pathogenesis of COVID-19 has been not fully explained, the data obtained so far in hospitalized patients has revealed that serum cytokine and chemokine levels are high in severe COVID-19 patients, similar to those found with sepsis [6–9]. Sepsis associated with COVID-19 is a topic that has recently been particularly emphasized [10]. Some researchers state that severe and critically ill patients meet the diagnostic criteria for sepsis and septic shock according to the Sepsis-3 International Consensus and recommend using the term ‘viral sepsis’ instead of the terms severe and critical illness because it is more appropriate [10,11]. 

## 2. Definition and etiology of sepsis

### 2.1. Definition

Sepsis is a severe potentially fatal clinical syndrome and is one of the leading causes of infection-associated death. Early recognition and effective management may provide improved outcomes [12]. The American College of Chest Physicians and the Society of Critical Care Medicine (SCCM) held a Consensus Conference on the definition of sepsis and published a paper in 1992, following which the definition of sepsis has been updated several times [3]. Sepsis is now defined as a life-threatening organ dysfunction caused by a dysregulated host response to infection in the 2016 update of the SSC guideline. Currently, the use of the sequential organ failure assessment (SOFA) score is recommended to identify both sepsis and septic shock. The SOFA score contains evaluation parameters pertaining to six organ systems including respiration, coagulation, liver, central nervous system, renal, and the circulatory system [1]. An acute change in total SOFA score ≥ 2 points according to the baseline assessment consequent to the infection is accepted as organ dysfunction, which is associated with an in-hospital mortality greater than 10%. In patients not known to have preexisting organ dysfunction, the baseline SOFA score is considered as zero. Septic shock is defined as the need for vasopressor therapy to maintain mean arterial pressure ≥ 65 mmHg despite adequate fluid resuscitation, and a lactate level >2 mmol/L in patients with sepsis. This condition has been reported to be associated with hospital mortality rates that may exceed 40% [13]. In 2016, a bedside clinical score, quick SOFA (qSOFA), was also developed. The qSOFA score consists of three parameters: respiratory rate of ≥22/min, altered mentation, and systolic blood pressure of ≤100 mm Hg. In ambulatory settings, emergency departments, or hospital wards, patients who are suspected to have sepsis can be easily evaluated utilizing the qSOFA score for poor outcomes. Patients who meet at least two of the criteria are considered candidates at risk for the development of sepsis and should be evaluated with the SOFA score.

In sepsis, two consecutive phases are seen, the initial phase being a hyper-inflammatory phase, which is then followed by an immunosuppressive phase [4]. There are numerous markers in the hyperinflammatory phase such as C-reactive protein (CRP), procalcitonin, tumor necrosis factors (TNF), interleukin (IL) 1β, and IL-6. TNF, IL-1β, and IL-6, which are pro-inflammatory cytokines and released as the initial response to injury or infection. CRP production is stimulated by IL-6 and is produced by the liver as a response to infection. Procalcitonin is produced in many cells of the body and considered to be the most useful marker severe systemic inflammation. Several of these markers are accepted as the biomarkers of sepsis and may be used to assist in the diagnosis and management of patients with sepsis [14]. Similarly, these markers have also been documented to be increased in patients with severe COVID-19 [6–8,10]. 

### 2.2. Etiology

Sepsis can develop secondary to bacteria, viruses, fungi, and other pathogens, with bacterial pathogens being most frequently implicated [15]. In a recent comprehensive study investigating sepsis etiology, the Extended Prevalence of Infection in Intensive Care II (EPIC II), the most common infection sites were determined to be the lungs (64%), abdomen (20%), and bloodstream (15%). The most common pathogens were gram-negative bacteria (62%), gram-positive cocci (47%), and fungi (19%) [16]. In another study evaluating community-acquired and hospital-acquired sepsis and septic shock, the most common infection sites were lungs, abdomen, and bloodstream in both groups. The most common pathogens isolated in this study included *Streptococcus pneumoniae,* *Escherichia coli,* and other gram-positive and gram-negative bacteria in community-acquired sepsis, and *Staphylococcus aureus*, nonfermentative, and other gram-positive and gram-negative bacteria in hospital-acquired sepsis [17].

In up to 42% of sepsis cases, no bacteria is isolated, suggesting that sepsis may be of a nonbacterial etiology. However, the proportion of viruses that have been implicated is very low, accounting for approximately 1% of reported sepsis cases [17]. However, recent reports emphasized that respiratuar viruses have been underestimated in sepsis and septic shock [18]. Influenza viruses, adenoviruses, and dengue viruses all have the capacity to cause viral sepsis. Influenza A and B viruses may cause severe infection in children younger than 5 years of age, adults, pregnant women, and immunocompromised individuals [15]. Herpes simplex viruses and enteroviruses result in viral sepsis in neonates. Other respiratory viruses including respiratory syncytial virus, coronaviruses, human metapneumovirus, parainfluenza virus types 1–3, adenovirus, enteroviruses, and rhinovirus can cause severe infections. Recent studies have reported the underestimation of sepsis cases caused by viruses [19,20]. Viral coinfections are common in respiratory infection and may also present with a clinical picture of sepsis but are often not considered or overlooked by clinicians. [20]. Severe infections associated with SARS-CoV-2 have sparked a debate or at least raised awareness about viral sepsis, and the viral etiology has become more acceptable as an important cause of sepsis.

## 3. Inflammation and cytokine storm in COVID-19 pathogenesis

### 3.1. Entry of the virus into cells and pathogenesis

**Figure 1:**
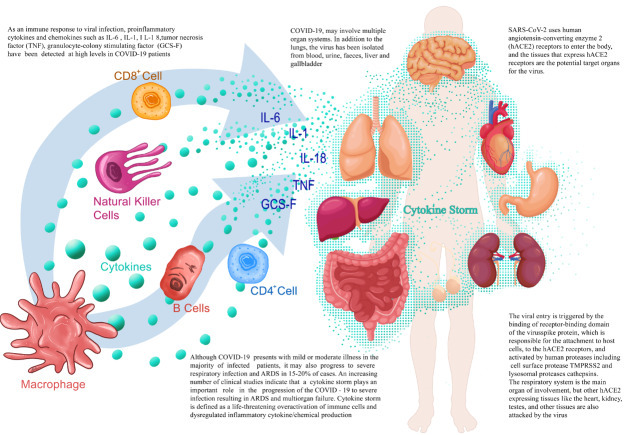
The cytokine storm and affected organs in COVID-19 sepsis.

COVID-19 may involve multiple organ systems. In addition to the lungs, the virus has been isolated from blood, urine, faeces, liver, and gallbladder [21,22]. The cell entry mechanism of the virus has been extensively researched in previous studies [23,24]. The viral entry, the involved organs and pathogenesis of severe COVID-19 are shown in Figure 1. SARS-CoV-2 uses human angiotensin-converting enzyme 2 (hACE2) receptors to enter the body, and the tissues that express hACE2 receptors are the potential target organs for the virus [25,26]. The viral entry is triggered by the binding of receptor-binding domain of the virus spike protein, which is responsible for the attachment to host cells, to the hACE2 receptors, and activated by human proteases including cell surface protease TMPRSS2 and lysosomal proteases cathepsins [23,27,28]. The respiratory system is the main organ of involvement, but other hACE2 expressing tissues like the heart, kidney, testes, and other tissues are also attacked by the virus [29]. The precise mechanisms involved in reaching and infecting other organs has not yet been fully elucidated. After entry of target cells, the virus releases its genome into the cytoplasm and initiates transcription. It suppresses the host immune response and enhances viral replication. In response to the virus, the host defenses are activated with presentation of major histocompatibility complex class I antigen. The host immune response results in either an effective control of the infection or an exaggerated immune response that may be deleterious to host tissues [30]. Toll-like receptors (TLRs), which are inflammatory receptors that are expressed in leukocytes, vascular endothelial cells, myocardiocytes, and alveolar type 2 cells are able to recognize pathogen components and can trigger hyperinflammation by inducing intracellular signaling systems such as chemokine and cytokines. It has been suggested that polymorphisms in the genes encoding TLRs may explain the varying spectrum of the immune response to SARS-CoV-2, ranging from the optimal response to exaggerated responses in COVID-19 patients [9].

Results from autopsy series in COVID-19 patients have demonstrated a wide range of findings including vascular involvement, congestion, consolidation, and hemorrhage as well as diffuse alveolar damage in lung tissue in keeping with acute respiratory distress syndrome (ARDS) [31]. The presence of viral cytopathic-like changes, infiltration of inflammatory cells (mononuclear cells and macrophages), and viral particles in histopathological samples are considered a consequence of both direct viral infection and immune hyperactivation. Furthermore, immunohistochemical staining has revealed that lung tissues such as the alveolar cavity and alveoli are infiltrated with CD20+B cells and CD8+T cells. The severe clinical pattern and the pathological and immunohistochemical alterations in the lung tissues suggest that overactivation of the immune system plays an important role in the lung lesions alongside direct viral damage [10,32].

Thromboembolism and hyper-coagulopathy are other components in the pathogenesis of severe COVID-19. Various studies have revealed that significant coagulation abnormalities occur in patients with COVID-19 and are correlated with disease severity [33–35]. The pathogenesis of COVID-19-associated coagulopathy is a complex series of events. Although the pathogenesis of hypercoagulability is not fully understood, it has been pointed out that all three components of Virchow’s triad (endothelial injury, stasis, and hypercoagulable state) play a major role in contributing to clot formation in severe COVID-19 infection [36,37]. Some researchers have proposed that severe COVID-19 is a microvascular disease in which the virus activates endothelial inflammation and injury. Hypercoagulability together with an inflammatory state occurs in many patients with severe infection, and this differs from acute DIC [37]. Thrombotic and hemorrhagic events have been reported as common complications in patients who have demised from SARS-CoV-2 infection. It has been reported that sepsis-induced coagulopathy typically occurs before disseminated intravascular coagulation, and sepsis-induced coagulopathy and disseminated intravascular coagulation scores have been reported to increase over time in patients who died [38]. 

### 3.2. Inflammation and cytokine storm

Although COVID-19 presents with mild or moderate illness in the majority of infected patients, it may also progress to severe respiratory infection and ARDS in 15%–20% of cases [39,40]. An increasing number of clinical studies indicate that a cytokine storm plays an important role in the progression of the COVID-19 to severe infection resulting in ARDS and multiorgan failure [41–43]. Cytokine storm is defined as a life-threatening overactivation of immune cells and dysregulated inflammatory cytokine/chemical production [32]. In severe COVID-19 cases, laboratory parameters such as hematological findings, coagulation tests, liver function tests, D-dimer, ferritin, and acute phase reactants such as CRP show marked alterations, which are suggestive of a cytokine storm [6,44]. As an immune response to viral infection, proinflammatory cytokines and chemokines such as IL-6, IL-1, IL-18, TNF, granulocyte-colony stimulating factor (GCS-F) have been detected at high levels in COVID-19 patients [6–8,10]. Other cytokines have been implicated as well. In addition, several publications have reported the detection of higher cytokine levels in severe COVID-19 patients as compared to nonsevere cases [8,40,43,45]. It has, therefore, been postulated and suggested that tissue injury leading to ARDS, and multiorgan failure in SARS-CoV-2 infection is mainly the result of cytokine overproduction. 

Another key element of COVID-19 pathogenesis in severe cases is its similarity or association with hemophagocytic lymphohistiocytosis (HLH). SARS-CoV-2 induced cytokine storm has significant clinical and laboratory findings overlapping with HLH. Fever, ARDS, cytopenia, the elevation of liver function tests, and ferritin elevations, which are common particularly in severe COVID-19, may have developed due to secondary HLH [8,38]. HLH is classified as primary HLH, which has a genetic inheritance and secondary HLH. Secondary HLH is associated with an underlying disease including, autoimmune disease, malignancy, and viral infections such as Epstein-Barr virus, cytomegalovirus, other herpes viruses, and SARS-CoV-2 [38,46]. 

In summary, it has been proposed that SARS-CoV-2 initiates a cascade of events that results in an overreaction of the immune system known as a cytokine storm or hyper-inflammation syndrome [47]. The exact mechanism of the cytokine storm, however, still needs to be fully elucidated. Although the source of cytokines is not exactly known, it has been stated that virus-infected cells such as type II pneumonocytes and endothelial cells may be implicated [30]. 

### 3.3. COVID-19 and Sepsis Link 

**Figure 2:**
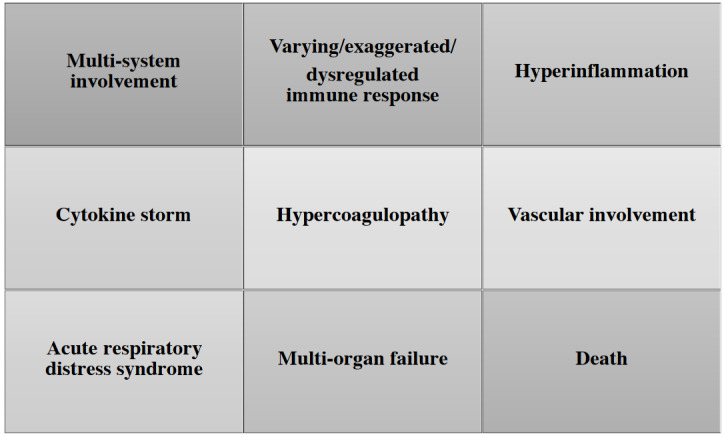
The clinical spectrum of sepsis in COVID-19.

From the outset of the pandemic, various reports have indicated that although there are some unique features pertinent to COVID-19, many of its acute manifestations are similar to sepsis caused by other pathogens [10,30,48,49]. The possible spectrum of sepsis in COVID-19 are shown in Figure 2. As has been well documented, patients with advanced age and underlying comorbidities such as hypertension, diabetes mellitus, chronic respiratory failure, heart disease and impaired immune status are at enhanced risk of more severe COVID-19, including ARDS, multiorgan failure, and death [39,44]. This severe form of COVID-19 disease has been related to the presence of a cytokine storm that is suggestive of sepsis [30,50]. Liu H et al. hypothesized that the process called viral sepsis is a crucial step in the mechanism of COVID-19 [10]. Others prefer the term ‘COVID-19 sepsis’ as many patients meet the diagnostic criteria for sepsis [10,50]. 

SARS-CoV-2 may cause sepsis regardless of secondary bacterial or fungal infections. Patil et al. have suggested that the virus itself likely causes a sepsis syndrome as a consequence of various possible mechanisms, including immune dysregulation, respiratory dysfunction leading to hypoxemia, and metabolic acidosis due to circulatory dysfunction. Multiorgan failure seen in COVID-19 could also be explained by hypoxia and circulatory disorders that occur secondary to microvascular dysfunction. Others have suggested that microvascular dysfunction may also contribute to hypoxia and subsequent organ failure through the interruption of blood flow to the lungs by disseminated intravascular coagulation and micro-embolism [30]. Lin HY emphasizes that various degrees of damage to the heart, liver, kidney, and other organs in severe infection together with laboratory abnormalities such as decreased lymphocyte and platelet counts, increased D-dimer, CRP, liver and myocardial enzyme levels, and high levels of cytokines are similar to those seen in sepsis caused by bacterial infections. He indicates that severe COVID-19 has all the hallmarks of sepsis including a specific pathogen and that COVID-19 should, therefore, be considered as sepsis caused by viral infection [49]. Other workers have reinforced this view, specifying that all infective pathogens, including viruses can cause sepsis [51]. Various viral respiratory pathogens, including influenza, avian and swine flu, severe acute respiratory syndrome (SARS), and Middle East respiratory syndrome (MERS) have been associated with sepsis [49].

Viral sepsis has some similarities but also some differences when compared to bacterial sepsis. In bacterial sepsis, systemic inflammation affecting multiple organs is more dominant than in COVID-19 sepsis [50]. While bacterial sepsis causes an early and sudden onset clinical deterioration, viral diseases may exhibit a relatively late onset and chronic course [49]. Systemic steroids when used appropriately, modulate the immune response and have been shown to improve survival in COVID-19 patients but are only recommended to be used to help reverse septic shock in bacterial sepsis Bhimraj A, Morgan RL, Shumaker AH, Lavergne V, Baden L et al. Infectious Diseases Society of America Guidelines on the Treatment and Management of Patients with COVID-19. Infectious Diseases Society of America 2021; Version 4.3.0. Available at https://www.idsociety.org/practice-guideline/covid-19-guideline-treatment-and-management/. Accessed 13 June 2021.[50,52]. Macrophage activation syndrome (MAS), also known as secondary HLH, is generally more common in viral infections [50].

Consideration of severe COVID-19 disease as a sepsis syndrome has relevance and may assist in terms of determining treatments that will modulate the immune response, limit intrinsic damage to tissue and organs, and potentially improve outcome.

## 4. SEPSIS THERAPY IN COVID-19

### 4.1. General Overview and Supportive Care

The SSC has issued a guideline on the management of critically ill patients in ICU with COVID-19 [53]. This guideline contains 54 statements, including various recommendations that cover aspects of infection control, laboratory diagnosis and specimens, hemodynamic support, ventilatory support, and COVID-19 therapy [53]. The SCC guideline has summarized its recommendations on treatment under three main headings, hemodynamics, ventilation, and therapy. The guideline has suggested using dynamic parameters (skin temperature, capillary refilling time, and/or serum lactate measurement) over static parameters to assess fluid responsiveness. Conservative fluid support with balanced crystalloids and using norepinephrine (noradrenaline) as the first-line vasoactive agent if needed, has been recommended. In the presence of COVID-19 and refractory shock, low-dose corticosteroid therapy (200 mg daily of hydrocortisone) is recommended.

**Figure 3:**
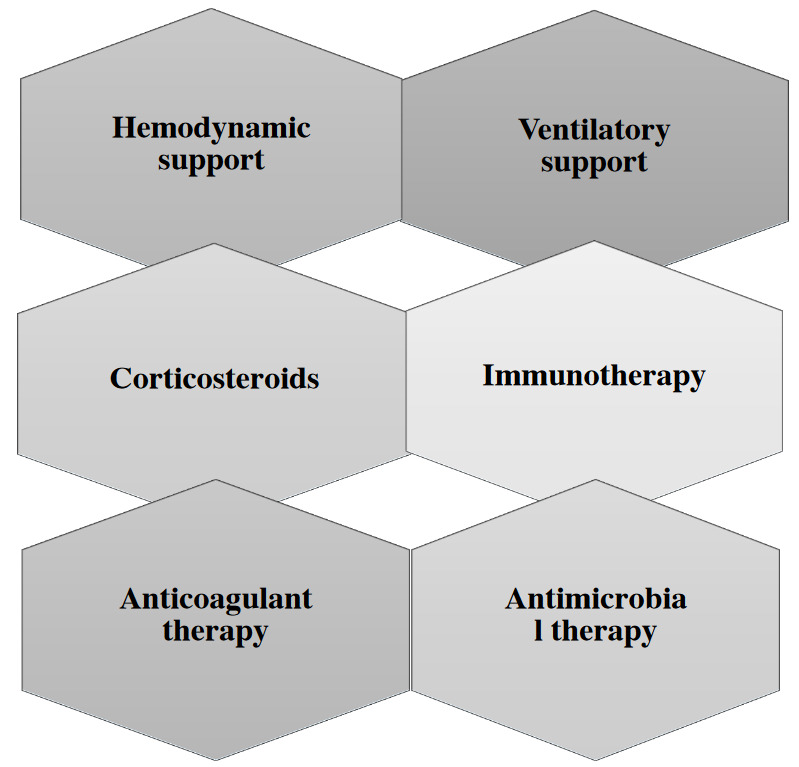
Therapeutical approach to severe COVID-19.

Supplemental oxygen is recommended if peripheral oxygen saturation is <92%. More than 75% of hospitalized patients require supplemental oxygen therapy, and some who do not respond to conventional oxygen therapy may require high-flow nasal cannula (HFNC) oxygen therapy [53]. The SCC guideline suggests use of HFNC and noninvasive positive-pressure ventilation (NIPPV) as options in adult COVID-19 patients with acute hypoxemic respiratory failure. Deciding when to initiate invasive mechanical ventilation in individuals with respiratory failure is determined by patient condition and characteristics [54]. Early and controlled intubation provides better management of some risks, such as minimizing nosocomial infections in healthcare workers [54]. Further and more detailed information on instituting and managing invasive mechanical ventilation is provided in the SSC guidelines on COVID-19 and the World Health Organization (WHO) guidelines on COVID-19^1^ [53]. The therapeutical approach to severe COVID-19 is summarized in Figure 3.

### 4.2. Specifics of therapy 

#### 4.2.1. Corticosteroids

At the beginning of the pandemic, systemic steroid treatment was not routinely recommended in the management of severe COVID-19 patients World Health Organization (2020). Clinical management of severe acute respiratory infection (SARI) when COVID-19 disease is suspected: interim guidance, 13 March 2020. World Health Organization. https://apps.who.int/iris/handle/10665/331446. License: CC BY-NC-SA 3.0 IGO. With subsequent better understanding and information about the pathogenesis of COVID-19, antiinflammatory therapies, especially corticosteroids, started to attract increasing global attention. At present, antiinflammatory therapies that suppress immune-mediated systemic inflammation and the cytokine storm play a key role in the treatment of severe COVID-19 patients. A metaanalysis on the use of corticosteroids in adult hospitalized patients with ARDS reported that corticosteroids may have potential benefits on mortality, ventilation duration, and ventilation-free days in patients with ARDS [55]. The RECOVERY Trial, a large, multicenter, randomized, and open-label study compared patients who received standard care with those who received dexamethasone for up to 10 days in addition to standard of care and found a lower mortality rate at 28 days in the dexamethasone arm of the study. This benefit was detected in only severe and critically ill COVID-19 patients and those who required supplemental oxygen. Patients not requiring supplemental oxygen therapy did not experience any benefit [56]. Based on this data, it is now accepted that corticosteroids are recommended in severe COVID-19 patients who also meet sepsis criteria. However, clinicians should be aware of recognized adverse effects of corticosteroids such as hyperglycemia, secondary infection, psychiatric disorders, and avascular necrosis.

Corticosteroids, preferably dexamethasone 6 mg IV or per os, or 50 mg of hydrocortisone intravenously every 8 h for up to 10 days or until hospital discharge (whichever comes first), are strongly recommended in severe and critically ill cases of COVID-19. Corticosteroids are not recommended in nonsevere COVID-19 cases/mild cases and in patients not requiring oxygen therapy [57]. The SCC guideline suggests the use of systemic steroid therapy in mechanically ventilated adults with ARDS [53]. 

#### 4.2.2. Immunotherapy 

With the understanding of the role of excessive immune response in the pathogenesis of severe COVID-19, specific antiinflammatory drugs that suppress the immune response in severe patients have been administered, and the experiences with these agents have been reported. Recent findings have shown the potential benefits of therapies used to suppress the cytokine storm such as anakinra and tocilizumab in secondary HLH (or Macrophage Activation Syndrome [MAS])^1^. Tocilizumab, an IL-6 receptor antagonist, has been a candidate drug for better survival in severe patients from the early days of the pandemic based on the evidence from studies in which IL-6 levels were found to be higher in severe patients compared to mild-to-moderate patients [44,58]. The RECOVERY trial reported a slight but statistically significant improvement in survival and other clinical outcomes with tocilizumab in COVID-19 patients with hypoxia and systemic inflammation as compared to standard of care [59]. The REMAP-CAP trial also reported similar results with the IL-6 antagonists tocilizumab and sarilumab in critically ill patients who required organ supportive care [60]. The recommended tocilizumab dose is 400 mg-800 mg IV (depending on weight). A second dose may be administered 12–24 h later in the patients whose condition has not improved [59]. Another potential effective antiinflammatory drug for severe COVID-19 is anakinra, an IL-1 inhibitor. IL-1 is a pro-inflammatory cytokine and associated with severe COVID-19. Several studies have reported improvements following anakinra treatment based on clinical outcomes, including reduction in mortality and reduced need for invasive mechanical ventilation. No increase in serious side effects have been reported [61–63]. 

Baricitinib, a selective Janus kinase inhibitor approved by the US Food and Drug Administration (FDA) for rheumatoid arthritis has also been investigated for COVID-19 treatment. It has both antiviral and antiinflammatory properties. A reduction in serum levels of IL-6, IL-1β, and TNF-α, rapid recovery of circulating T and B cell frequencies, and increased antibody production against the SARS-CoV-2 spike protein has been reported in severe COVID-19 cases treated with this agent [64]. Baricitinib has been advocated in select severe COVID-19 cases having elevated inflammatory markers and who are not receiving invasive mechanical ventilation, based on data showing a reduction in 28-day mortality. The recommended dose is 4 mg daily dose for 14 days or until discharge from the hospital^1^. 

#### 4.2.3. Anticoagulant therapy 

Thromboembolism, one of the potential pathogenetic mechanisms in the development of COVID-19 sepsis, is common in COVID-19 cases, particularly in severe cases. To date, various guidelines have addressed anticoagulant therapy in COVID-19 COVID-19 Treatment Guidelines Panel. Coronavirus Disease 2019 (COVID-19) Treatment Guidelines. National Institutes of Health. Available at https://www.covid19treatmentguidelines.nih.gov/. Accessed [13 June 2021]. [65–67]. Anticoagulant prophylaxis is recommended for all hospitalized COVID-19 patients unless contraindicated^3^. Most guidelines advocate prophylactic anticoagulant drug dosing^3 ^[65–66]. Options for thromboprophylaxis include low molecular weight heparin (LMWH) or unfractionated heparin, if LMWH is unavailable. Enoxaparin is usually administered in a dose of 40 mg by subcutaneous injection daily, with dose adjustment for body weight and renal dysfunction. Tinzaparin, dalteparin, and fondaparinux are the other potential agents that may be used for thromboprophylaxis. The suggested duration of thromboprophylaxis is until discharge. Patients who are already receiving a therapeutic dose of anticoagulant treatment should continue on this dose of anticoagulant therapy [57]. 

#### 4.2.4. Antimicrobial therapy

Viral sepsis is similar to bacterial sepsis in terms of clinical appearance, laboratory and cytokine parameters, and its treatment principles revolve around appropriate supportive therapy, immune regulatory therapy, and possibly effective antiviral treatment. Antibiotics should not be routinely administered in COVID-19 cases. At the beginning of the pandemic, a few small studies reported up to 95% high coinfection rates with bacterial, fungal, and other viral pathogens [68–69]. However, subsequent larger studies reported much lower coinfection rates. In an observational study from the U.S, coinfection was observed in only 3.6% of hospitalized COVID-19 patients, whilst 79% of these patients had received antibiotics [70]. In hospitalized COVID-19 patients, antibiotic overuse is estimated to have contributed to an increase in antibiotic resistance during the COVID-19 pandemic [71]. Current guidelines recommend that antibiotics not be routinely prescribed in suspected and confirmed COVID-19 patients in which there is a low suspicion of a bacterial infection in order to avoid unnecessary adverse effects and antimicrobial resistance^3^ [57]. In the WHO living guideline on COVID-19, the use of empirical antibiotic therapy for likely pathogens is recommended in cases of severe COVID-19, based on astute clinical judgment and local epidemiology. In this setting, antimicrobial therapy should be commenced early (ideally within 1 h) and duration should be as short as possible (5–7 days) [57].

#### 4.2.5. Other therapies

Several treatment options have been investigated in severe COVID-19. In light of the data obtained from previous studies, routine intravenous immunoglobulins (IVIGs) and convalescent plasma should not be used in critically ill patients [53]. Blood filtration devices have been advocated as a potential therapeutic approach in severe COVID-19 infection. Although viruses are not killed or destroyed by these devices, inflammatory mediators induced by viruses and that may be potentially harmful can be absorbed and removed from the body [72–73]. Further studies investigating the benefit of these treatments in severe COVID-19 patients are needed, however, to make precise recommendations. 

## 5. Conclusion

A considerable effort has been made since the beginning of the pandemic to understand COVID-19, and much progress has been made regarding its pathogenesis, epidemiology, and clinical features. There are, however, still some unclear points, particularly relating to the pathogenesis of severe illness. Evidence obtained from clinical and autopsy studies indicates that direct viral damage, infiltration of organs with immune cells, and excessive production of cytokines are responsible for the pathology in severe COVID-19. This situation is similar to sepsis syndromes caused by other pathogens. Many COVID-19 patients meet the diagnostic criteria for sepsis, and, consequently, the basic elements and principles of the treatment approach in COVID-19 patients should be addressed in a similar fashion to other sepsis patients. Immunotherapies appear to play a relevant role in the treatment of severe cases of COVID-19 consequent to an exaggerated immune response, and a wealth of experience has accumulated over the past two years regarding their use in COVID-19 sepsis. We will continue to learn more over time.
